# Confidence in visual detection, familiarity and recollection judgments is preserved in schizophrenia spectrum disorder

**DOI:** 10.1038/s41537-023-00387-4

**Published:** 2023-09-07

**Authors:** Martin Rouy, Michael Pereira, Pauline Saliou, Rémi Sanchez, Wassila el Mardi, Hanna Sebban, Eugénie Baqué, Childéric Dezier, Perrine Porte, Julia Micaux, Vincent de Gardelle, Pascal Mamassian, Chris J. A. Moulin, Clément Dondé, Paul Roux, Nathan Faivre

**Affiliations:** 1 Univ. Grenoble Alpes, Univ. Savoie Mont Blanc, CNRS, LPNC, 38000 Grenoble, France; 2 Centre Hospitalier de Versailles, Service Hospitalo-Universitaire de Psychiatrie d’Adultes et d’Addictologie, Le Chesnay; Université Paris-Saclay; Université de Versailles Saint-Quentin-En-Yvelines; DisAP-DevPsy-CESP, INSERM UMR1018, Villejuif, France; 3 Centre d’Économie de la Sorbonne, CNRS and Paris School of Economics, Paris, France; 4 Laboratoire des Systèmes Perceptifs, Département d’Études Cognitives, École Normale Supérieure, PSL University, CNRS, Paris, France; 5 Univ. Grenoble Alpes, Inserm, U1216, Adult Psychiatry Department CHU Grenoble Alpes, Grenoble Institut Neurosciences, 38000 Grenoble, France; 6 Adult Psychiatry Department, CH Alpes-Isère, F-38000 Saint-Egrève, France

**Keywords:** Schizophrenia, Human behaviour

## Abstract

An effective way to quantify metacognitive performance is to ask participants to estimate their confidence in the accuracy of their response during a cognitive task. A recent meta-analysis^1^ raised the issue that most assessments of metacognitive performance in schizophrenia spectrum disorders may be confounded with cognitive deficits, which are known to be present in this population. Therefore, it remains unclear whether the reported metacognitive deficits are metacognitive in nature or rather inherited from cognitive deficits. Arbitrating between these two possibilities requires equating task performance between experimental groups. Here, we aimed to characterize metacognitive performance among individuals with schizophrenia across three tasks (visual detection, familiarity, recollection) using a within-subject design while controlling experimentally for intra-individual task performance and statistically for between-subject task performance. In line with our hypotheses, we found no metacognitive deficit for visual detection and familiarity judgments. While we expected metacognition for recollection to be specifically impaired among individuals with schizophrenia, we found evidence in favor of an absence of a deficit in that domain also. We found no specific metacognitive deficit in schizophrenia spectrum disorder in the visual or memory domain. The clinical relevance of our findings is discussed in light of a hierarchical framework of metacognition.

## Introduction

Confidence abnormalities in the form of overconfidence in errors in schizophrenia spectrum disorders have been documented in multiple cognitive domains, including memory, perception, and emotion recognition^2^. Yet, the hierarchical level at which these abnormalities occur is still unclear. In line with the terminology proposed by Galvin and colleagues^3^, cognitive performance is referred to as first-order performance (i.e., how well one is able to detect or discriminate between probed stimuli), and metacognitive performance is referred to as second-order performance (i.e., how well one is able to discriminate between correct and incorrect responses). Properly quantifying metacognitive performance requires controlling for variations of cognitive performance that are not metacognitive in nature^3–5^. This concern is of particular relevance in schizophrenia, where cognitive deficits are well documented^6,7^. In a meta-analysis that was recently conducted^1^, metacognitive processes pertaining to perception (i.e., metaperception) were mostly preserved when first-order performance was controlled for. Yet, conclusions about metacognitive processes pertaining to memory (i.e., metamemory) could not be drawn in this meta-analysis since the medium to large effect size deficit resulted from studies where memory performance was not equated between patients and healthy controls (except for one study^8^). In these conditions, metamemory deficits were likely to be confounded with memory deficits. More recently, a study by Zheng and colleagues^9^ controlling for first-order performance reported that individuals with schizophrenia discriminated correct memory decisions from incorrect ones as accurately as the controls.

Here, we sought to establish if schizophrenia is characterized by metacognitive deficits in the perceptual and memory domains irrespective of first-order performance for two main reasons. At the clinical level, we considered that drawing a finer map of metacognitive deficits in schizophrenia is important to better track the origins of the lack of insight among patients^10^ and to guide metacognitive remediations that are now used therapeutically^11^. At the fundamental level, we reasoned that quantifying how deficits in metaperception and metamemory covary would help to determine whether metacognition is implemented following a domain-general or domain-specific architecture^12^.

To compare metaperceptual and metamemory deficits in individuals with schizophrenia while controlling for perceptual and memory deficits, we developed a novel experimental paradigm including three randomly interleaved perceptual and memory tasks attempting to experimentally match first-order performance at the intra-individual level across tasks and to statistically control for performance at the inter-individual level.

We preregistered our main predictions based on current knowledge regarding the cognitive architecture of perception and memory and their impairments in schizophrenia (see ref. ^13^ for a review). Individuals with schizophrenia typically have preserved performance in familiarity judgments (i.e., decontextualized memory^14^) but impaired performance in recollection judgments (i.e., episodic/recollection memory necessitating multimodal integration via hippocampal activity^15^), which may be explained by impaired hippocampus recruitment^16^ and hippocampal atrophy^17^. Our main preregistered hypothesis was that metamemory was globally more impaired than metaperception, assuming that previous reports of deficits in metamemory were not only driven by deficits taking place at the first-order level and considering that recollection and perception may involve distinct metacognitive processes^18^. Furthermore, since familiarity can be considered a perceptual-mnemonic process storing decontextualized perceptual elements^19^, we hypothesized domain-generality between perception and familiarity processes and meta-recollection to be specifically impaired. Besides this preregistered hypothesis, we explored the links between metacognitive performance and clinical traits such as positive, negative and disorganization syndromes.

## Methods

### Participants

Following a preregistered open-ended sequential Bayes Factor design (see Supplementary Information), we recruited 38 individuals with schizophrenia and 39 healthy control participants matched for age, sex, education level and premorbid IQ (see Table 1 for demographic and clinical information). After exclusions according to preregistered criteria (essentially due to ceiling performance, see Supplementary Information), the analyses were conducted on a sample of 34 individuals with schizophrenia and 36 healthy controls. Two licensed psychiatrists (CD and PR) confirmed the diagnoses in the schizophrenia group according to the DSM-V criteria for schizophrenia (details about the recruitment procedure are provided in Supplementary Information). All participants were naive to the purpose of the study, gave written informed consent in accordance with institutional guidelines and the Declaration of Helsinki, and received monetary compensation (10€/h) except those participants under legal protection. The study was approved by the ethical committee *Sud Méditerranée* II on April 3, 2020 (217 R01 MS1).

**Table 1:** Sociodemographic and clinical characteristics of individuals with schizophrenia and control participants.

	Control *N* = 36 (mean ± SD)	Schizophrenia *N* = 34 (mean ± SD)	t-statistic	*p*-value	Bayes factor
Age, years	34.5 ± 14.3	38.3 ± 11.1	1.26	0.21	0.48
Education level, years	12.9 ± 1.3	12.8 ± 2.7	−0.16	0.87	0.26
Premordid IQ	108.2 ± 6.8	104.9 ± 12.5	−1.30	0.20	0.55
WAIS Matrix subtest	9.6 ± 2.9	8.4 ± 2.5	−1.87	0.07	1.07
Calgary Depression Scale, score	1.5 ± 1.7	4.7 ± 3.9	4.20	<0.001	209.00
Illness duration, years		14.7 ± 9.3			
BCIS, composite score		8.2 ± 6.2			
SSTICS, total		30.1 ± 16.1			
SSTICS, working memory		4.6 ± 2.7			
PANSS, positive		13.2 ± 6.3			
PANSS, negative		11.7 ± 9.8			
PANSS, disorganization		21.7 ± 7.8			
PANSS, total		48.8 ± 33.7			

*p*-values are not corrected for multiple comparisons. Bayes factors are based on Bayesian t-tests with a scaling factor of 0.7.

*WAIS* Wechsler Adult Intelligence Scale (standardized scores), *BCIS* Beck Cognitive Insight Scale, *SSTICS* Subjective Scale To Investigate Cognition in Schizophrenia, *PANSS* Positive And Negative Symptoms in Schizophrenia.

### Experimental design

A video description of each task is available online (https://gitlab.com/nfaivre/metaface_scz_public/-/tree/main/videos).

#### Stimuli

Each face (see Supplementary Information for details) was presented against a visual background noise consisting of its phase-scrambled version. The background was colorized in blue or red (balanced for luminosity) to provide a contextual cue.

#### Procedure

##### Memory tasks

**Fig. 1:**
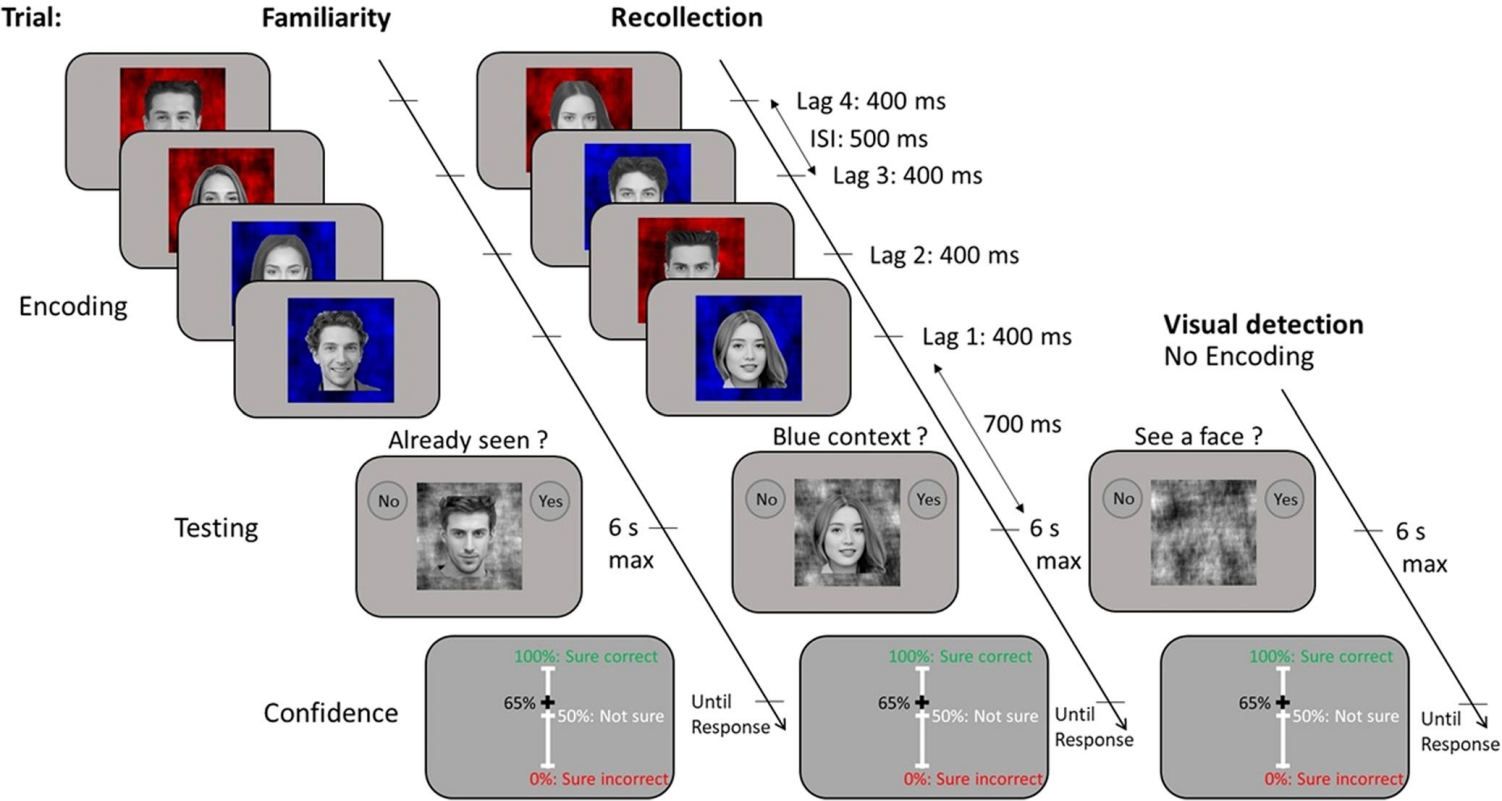
Experimental design. Timeline of the familiarity, recollection and visual detection tasks. The timeline was identical in the familiarity and recollection task, except for the testing phase where the question was task-specific: “Already seen?” for familiarity, and “Blue context?” for recollection. No encoding took place in the visual detection task. In the present illustration, the correct answers to the familiarity, recollection and perceptual questions are respectively: “No”, “Yes”, and “No”. Lag is an ordinal variable corresponding to the temporal distance between the target stimulus and the test stimulus.

The familiarity and recollection tasks shared the same timeline (Fig. 1). Each trial started with an encoding phase consisting of four successive face stimuli presented during 400 ms each (random combination of 2 male and 2 female faces) on a blue or red background (context), with a 500 ms inter-stimulus interval. To avoid learning effects and familiarity confounds, each face was presented only once throughout the whole experiment. Following the encoding phase, the test phase consisted in presenting a fifth face on a gray background and asking a task-specific question. In familiarity trials, the participant was asked to indicate whether the face had already been seen (80% of the trials, to obtain a uniform distribution across “stimulus strength” levels, see next paragraph) or not (20% of the trials); in recollection trials, the fifth face was always a seen face (i.e., a face presented during the encoding phase), and the participant was asked whether the context of this stimulus was blue (80% of the trials) or not (20% of the trials) during the encoding phase. Participants provided their answers with a mouse click on the “no” or “yes” buttons, respectively displayed at the top left and top right of the screen.

The difficulty of the familiarity and recollection tasks was manipulated by changing the serial position of the target stimulus during the encoding phase. Accordingly, there were four levels of stimulus strength—ranging from 1 to 4—corresponding to each of the four faces displayed sequentially within the encoding phase. Because this variable corresponds to the temporal distance between the target stimulus and the test stimulus, we refer to it as a “lag” (see Fig. 1 and Supplementary Information).

##### Visual detection task

Participants had to indicate whether a face was present (80% of the trials) or not (catch trials: 20%). The face could be presented at four contrast levels, chosen to match performances obtained in the memory tasks for each of the four lags (see Supplementary Fig. S2D). A fifth level—stimulus strength 0—was used to tag catch trials: trials where no face was presented (20% of the trials). As for memory trials, participants provided their answers with a mouse click on the “no” or “yes” buttons displayed at the top left and top right of the screen.

##### Confidence rating

For all three tasks, participants were asked to provide confidence judgments. After each first-order response (i.e., responses given to the familiarity, recollection and visual detection tasks), participants were asked to report their subjective confidence regarding the correctness of their decision by moving a slider with the mouse on a visual analog scale (see Fig. 1) ranging from 0% (“Sure incorrect”) to 100% (“Sure correct”). The initial position of the cursor for each trial corresponded to 50% confidence (“Not sure”).

#### Structure of the experiment

This protocol aimed to match intra-individual performance across familiarity, recollection, and visual detection tasks. Participants were asked to perform two sessions of 1 h each. Session 1 allowed us to measure memory performance at four difficulty levels (according to the variable “lag”, see “Memory Tasks” section*)*. We then matched perceptual performance to memory performance by determining four adequate contrast levels for the visual detection task for each participant (see Supplementary Information for details). Thus, session 1 provided four levels of stimulus strength, i.e., 4 memory lags and 4 visual contrast levels, corresponding to matched performance for each participant. Based on these individual parameters, session 2 contained 10 blocks of 30 randomly interleaved trials (familiarity, recollection and visual detection task), totalizing 300 trials (100 trials per task), each followed by a confidence rating task. Task order and stimulus strength were randomized, so participants could not predict which task they were going to perform on each trial.

Importantly, this paradigm was designed to match first-order performance between tasks, which is convenient for comparing metacognitive deficits across tasks. Although we also attempted to match first-order performance between groups, pilot experiments revealed this was not possible using adaptive staircases. Therefore, differences in task performance between groups were accounted for at the statistical level using the confidence efficiency metric^20^, taking advantage of our design with different levels of difficulty.

### Sociodemographic and neuropsychological characterization

The groups’ sociodemographic (age, sex, education), neuropsychological (National Adult Reading Test measuring patients’ premorbid IQ^21^, matrix reasoning subtest from the Wechsler Adult Intelligence Scale version IV^22^ and mood (Calgary Depression Scale^23^) characteristics were compared using the Student *t*-test or *χ*^2^ test when appropriate. Patients were characterized in terms of cognitive insight (using the self-reported Beck Cognitive Insight Scale^24^), schizophrenia symptomatology (using the clinician-evaluated Positive And Negative Syndrome Scale^25^, with factorial scores^26^) and subjective evaluation of cognitive functioning (using the self-reported Subjective Scale To Investigate Cognition in Schizophrenia^27^). As additional analyses, we explored whether metacognitive performance was correlated with demographic characteristics and clinical scores.

### Metacognitive performance

Here, metacognitive performance refers to the ability to adapt confidence to first-order performance. It was quantified by confidence efficiency and metacognitive sensitivity. Confidence efficiency relies on an explicit generative model of confidence, providing population-level estimates that can account for potential differences in first-order performance. Metacognitive sensitivity is a measure of the relationship between first-order accuracy and confidence (via individual regression slopes) obtained from Bayesian mixed-effects logistic regressions.

#### Bayesian mixed-effects logistic regressions

We conducted two Bayesian mixed-effects logistic regressions on first-order accuracy as a function of standardized confidence, group, stimulus strength, and task. Results were interpreted on the basis of the Bayes factor (BF) which reflects the relative evidence between the alternative and the null hypothesis according to Wagenmakers and colleagues^28^. Models formulae are provided in Supplementary Information.

#### Confidence efficiency

As preregistered, we assessed metacognitive performance while accounting for first-order performance and task difficulty with a recently developed metacognitive index called “confidence efficiency”^20^, here adapted to confidence ratings. This index is based on a generative model of confidence judgments based on Signal Detection Theory, where observers’ confidence judgments are not only subject to metacognitive noise but may also incorporate additional information from the stimulus. Interestingly for us, this method enables the simultaneous modeling of confidence responses across different levels of task difficulty, unlike other methods such as M-ratio^4,5^.

We estimated confidence efficiency by collapsing all participants into one global population after normalizing for variations in task performance across individuals, and we quantified its dispersion using a bootstrapping procedure. Namely, we computed 1000 confidence efficiency estimates based on a random resampling of our pool of participants (with replacement), resulting in one estimation distribution per task and group.

Our predictions regarding metacognitive performance (i.e., confidence efficiency and metacognitive sensitivity) were as follows: (1) A metamemory deficit for individuals with schizophrenia compared to healthy controls. (2) A significant interaction effect between group and task reflects a larger deficit in recollection metamemory among individuals with schizophrenia compared to other tasks, whereas healthy controls show no differences in metacognitive performances across tasks.

We also expected intra-individual first-order performances to be matched (assessed with model 1a, see Supplementary Information), as reflected by equivalent accuracy across the three tasks among patients and healthy controls. Since we did not experimentally adapt task performance between groups, we expected lower task performances among patients compared to controls.

## Results

### Clinical and neuropsychological variables

Groups were balanced for sex (χ^2^ = 0.25, *p* = 0.62) and comparable for age, education level, premorbid IQ, and scores on the WAIS matrix subtest (Table 1). However, individuals with schizophrenia had higher depression scores (mean ± SD: 4.7 ± 3.9) than healthy controls (mean ± SD: 1.5 ± 1.7, t = 4.20, *p* < 0.001, BF = 209). Descriptive statistics regarding false alarms, hits and confidence are described in Table 2 and show that in both groups, participants were performing all tasks correctly (i.e., better than chance).

**Table 2:** Experimental characteristics of individuals with schizophrenia and healthy control participants.

	Task	Control *N* = 36 (mean ± SD)	Schizophrenia *N* = 34 (mean ± SD)	t-statistic	*p*-value	Bayes factor
% False alarms	Visual detection	20.0 ± 20.7	16.9 ± 24.5	−0.57	0.57	0.28
	Familiarity	17.1 ± 14.1	24.6 ± 21.1	1.74	0.09	0.92
	Recollection	27.9 ± 17	50.6 ± 23.3	4.63	0.00	1211.87
% Hits	Visual detection	80.2 ± 13	70.6 ± 18.4	−2.50	0.02	3.53
	Familiarity	81.3 ± 10.4	72.8 ± 20.1	−2.20	0.03	2.02
	Recollection	78.5 ± 15.9	72.3 ± 14.9	−1.68	0.10	0.82
% Confidence in errors	Visual detection	82.8 ± 11.5	85.6 ± 11.4	1.02	0.31	0.39
	Familiarity	73.1 ± 12	72.7 ± 11.2	−0.13	0.89	0.25
	Recollection	73.2 ± 12.1	70.5 ± 11	−0.97	0.33	0.37
% Confidence in correct responses	Visual detection	95.0 ± 5.3	91.6 ± 9.7	−1.82	0.07	1.06
	Familiarity	89.2 ± 6.8	84.6 ± 11	−2.07	0.04	1.57
	Recollection	87.4 ± 10	80.9 ± 10.9	−2.58	0.01	4.04

*p*-values are not corrected for multiple comparisons. Bayes factors are based on Bayesian t-tests with a scaling factor of 0.7.

### First-order performance

**Fig. 2:**
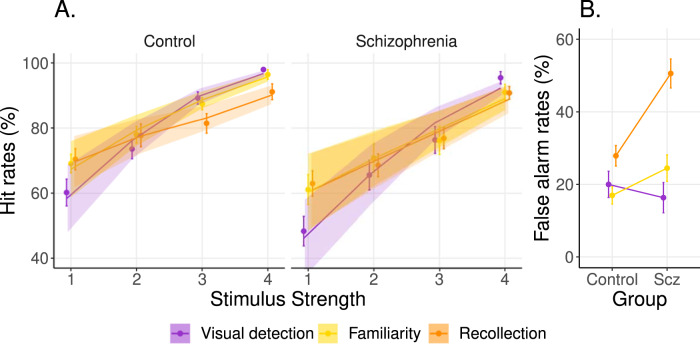
First-order performance. **A** Hit rates (i.e., rates of “yes” responses following stimuli with stimulus strength >0) across stimulus strengths in the visual detection (purple), familiarity (yellow), and recollection tasks (orange). Points and error bars indicate average accuracy and standard error of the mean, respectively; solid lines and shaded areas represent model fit mean and 95% confidence interval, respectively. **B** False-alarm rates (i.e., rates of “yes” responses following stimuli with 0 stimulus strength) across groups. Points and error bars indicate the average accuracy and standard error of the mean, respectively.

Model 1a revealed that patients had lower performance than healthy controls in the visual detection, familiarity and recollection tasks, and these first-order deficits were similar across tasks (i.e., no first-order interactions, see Table 3, Fig. 2A).

**Table 3:** First-order deficits across tasks.

		Estimate [95% CrI]	Bayes Factor
First-order deficits	Schizophrenia-Control (Visual detection)	−0.82 [−1.43, −0.21]	8.40
	Schizophrenia-Control (Familiarity)	−0.81 [−1.49, −0.13]	3.07
	Schizophrenia-Control (Recollection)	−0.62 [−1.27, 0.02]	1.23
First-order interactions	Group x task (Familiarity–Visual detection)	0.01 [−0.57, 0.62]	0.31
	Group x task (Recollection–Visual detection)	0.2 [−0.38, 0.78]	0.38
	Group x task (Recollection–Familiarity)	0.19 [−0.41, 0.81]	0.27
Intra-individual performance-matching	Familiarity–Visual detection (Control)	0.13 [−0.31, 0.56]	0.26
	Recollection–Visual detection (Control)	−0.2 [−0.62, 0.21]	0.32
	Recollection–Familiarity (Control)	−0.33 [−0.78, 0.12]	0.50
	Familiarity–Visual detection (Schizophrenia)	0.14 [−0.31, 0.58]	0.20
	Recollection–Visual detection (Schizophrenia)	0 [−0.44, 0.43]	0.16
	Recollection–Familiarity (Schizophrenia)	−0.14 [−0.59, 0.31]	0.14

We report posterior distributions’ summary statistics (mean and 95% credible interval) along with Bayes factors.

Differences in performance were expected as task performance was not experimentally controlled between groups. However, our procedure was designed to match intra-individual performance across tasks. Accordingly, pairwise first-order task performances were similar among patients and among control participants (Table 3). This confirms that our procedure globally matched intra-individual performance across tasks, although it did not match intra-individual performance for each stimulus strength (see Supplementary Table S1).

Patients and controls were sensitive to task manipulation of stimulus strength, as indicated by a strong effect of stimulus strength in all tasks (See Supplementary Table S1 and Fig. 2A).

Compared to healthy controls, patients had a similar false alarm rate in both the visual detection task (i.e., reporting seeing a face when none was presented: −0.22 [−1.02, 0.57], BF = 0.45) and in the familiarity task (i.e., reporting having seen the test face during the encoding phase when presented with a new face: 0.78 [−0.07, 1.64], BF = 2.21) but they committed significantly more false alarms in the recollection task (i.e., reporting having seen the test face in a given context during the encoding phase when presented in another context: 1.54 [0.61, 2.46], BF = 96.7) (Fig. 2B). The correlation between first-order reaction times and confidence known to be consistent across tasks^29^, but degraded in schizophrenia^30^ is described in Supplementary Information.

### Second-order performance

#### Confidence

Confidence levels were similar between patients and controls, except for the recollection task where patients were underconfident in correct responses (Table 2, confidence mean ± SD: 80.9 ± 10.9) compared to controls (confidence mean ± SD: 87.4 ± 10.0, t = −2.58, *p* < 0.05, BF = 4.04).

#### Metacognitive sensitivity

**Fig. 3:**
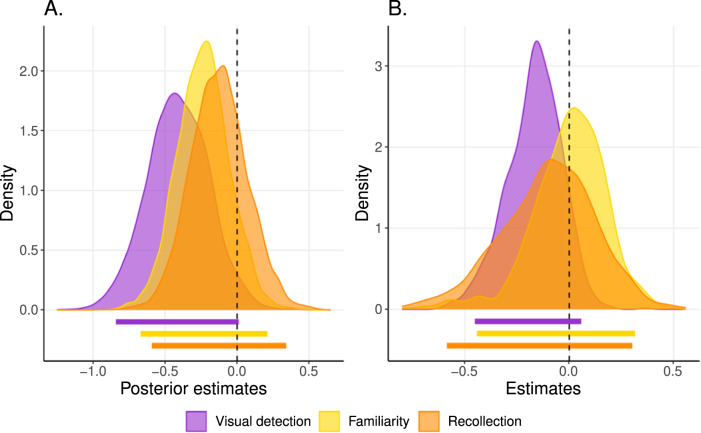
Second-order performance. **A** Bayesian posterior distributions of differences of regression slope estimates between patients and controls (i.e., distributions of metacognitive deficits estimations): meta-perceptual difference (purple), meta-familiarity difference (yellow), meta-recollection difference (orange). Vertical dashed line (estimate = 0) represents no difference between patients and controls. Horizontal colored bars indicate 95% credible intervals. **B** Distributions of differences in confidence efficiency estimates between patients and controls. Horizontal colored bars indicate 95% confidence intervals.

When quantifying metacognitive sensitivity as the slope between accuracy and confidence in mixed-effects logistic regressions (model 1a), individuals with schizophrenia were not found to underperform compared to healthy controls (Fig. 3A). Although qualitatively, the results could suggest a metacognitive deficit in the visual detection task, the evidence was statistically inconclusive (−0.41 [−0.84, 0.01], BF = 1.33). By contrast, we obtained moderate evidence in favor of an absence of a deficit both in meta-familiarity (−0.24 [−0.59, 0.12], BF = 0.32) and meta-recollection (−0.13 [−0.51, 0.27], BF = 0.17). Moreover, there was no difference of deficit between tasks (Familiarity–Recollection: 0.11 [−0.33, 0.56], BF = 0.18); Familiarity–Perception: 0.17 [−0.26, 0.62], BF = 0.28; Recollection–Perception: 0.28 [−0.18, 0.75], BF = 0.47). As discussed above, metacognitive sensitivity can be contaminated by differences in terms of first-order performance, which was only partially controlled in our paradigm. To estimate metacognitive performance independently of first-order performance, we turned to another metric called confidence efficiency.

#### Confidence efficiency

When quantifying metacognitive performance using the confidence efficiency measure of metacognition—which controls for first-order deficits—individuals with schizophrenia had similar confidence efficiency to the control group in the detection (−0.17 [−0.45, 0.06]), familiarity (−0.00 [−0.44, 0.31]) and recollection tasks (−0.10 [−0.58, 0.30]) (Fig. 3B). Within each group, confidence efficiency was comparable across tasks (Controls: Visual detection–Familiarity: −0.11[−0.40, 0.20], Visual detection–Recollection: −0.18[−0.51, 0.17], Recollection–Familiarity 0.07[−0.26, 0.41]; Patients: Visual detection–Familiarity: −0.28[−0.55, 0.14], Visual detection–Recollection: −0.24[−0.57, 0.19], Recollection–Familiarity −0.03[−0.50, 0.42]).

Contrary to some of our predictions about domain-generality, metacognitive performance as measured with mixed-effects logistic regressions did not correlate across tasks, neither did we find evidence for significant correlations with clinical traits such as positive, negative and disorganization syndromes and cognitive insight (see Supplementary Figs. S7 and S8).

## Discussion

The present study aimed at characterizing metamemory and metaperception in people with schizophrenia while controlling for first-order deficits, extending previous attempts in typical human samples^31^ and nonhuman primates^32^. In particular, we assessed metacognition in visual detection, familiarity, and recollection tasks. We hypothesized that people with schizophrenia would be specifically impaired in the meta-memory domain. At the first-order level, we found that people with schizophrenia had lower first-order performance in the three tasks compared to healthy controls. Contrary to our hypothesis, we found that metacognitive sensitivity was preserved among individuals with schizophrenia in the three tasks. In what follows, we discuss technical and conceptual aspects of our paradigm that should be considered to interpret this result and then examine its clinical and theoretical significance.

A key contribution of this study is our attempt to match first-order performance between tasks for each participant using adaptive procedures and between groups of participants using a generative model of confidence. We note that our adaptive procedure to match performance between tasks was successful when considering average performance but not when considering task performance across levels of stimulus strength. In other words, we equated the overall performance but not the slopes between tasks in Fig. 2A (see Supplementary Information for details). A plausible explanation for this is a contextual effect. In session 1, blocks of visual detection trials were separated from blocks of memory trials, whereas in session 2, the three tasks were interleaved within each block of trials. Thus, the visual detection psychometric curve (Supplementary Fig. S2c) from which we determined four visual contrast levels were obtained from a sequence of low-contrast perceptual stimuli (3 × 80 stimuli in a row), whereas during session 2, these low-contrast visual stimuli were interleaved with high-contrasted memory stimuli. This contextual effect might have resulted in a rightward shift (See Supplementary Fig. S3) of the visual detection psychometric curve, leading to underperformance in both groups in the visual detection task compared to the familiarity and recollection tasks.

Regarding between-group comparisons, the two groups were matched on age, education, premorbid IQ, and perceptual verbal reasoning. In contrast, depressive symptomatology was also higher in patients than in controls in the current study. Early pilot versions of the present protocol aimed at equating memory performance between participants using adaptive staircases that manipulated either the number of encoding items or the lag variable, but these attempts were not successful (no convergence). Instead, we accounted for differences in task performance between groups by relying on measures of confidence efficiency from a recent generative model of confidence^20^, which enables the estimation of metacognitive performance in factorial designs. Although this framework is recent and has not been fully benchmarked yet, we note that we found qualitatively similar results using a Bayesian logistic mixed-effects regression, which does not consider possible cognitive deficits but has the advantage of providing hierarchical estimates of metacognitive sensitivity, dealing with unbalanced data, and considering prior knowledge to compute Bayes factors. In contradiction to existing literature, both frameworks revealed no evidence for a metacognitive deficit in any of the three tasks. In fact, we found evidence for an absence of metacognitive deficit in memory tasks and only inconclusive evidence in the perceptual domain. We acknowledge that our results do not provide a clear picture regarding the importance of controlling for first-order performance, as both measures of metacognitive performance yielded similar conclusions. Such control may be useful in other contexts notably to account for compensation mechanisms resulting in preserved metacognitive performance in the presence of cognitive deficits.

The absence of metacognitive deficit in schizophrenia was corroborated by an absence of difference regarding confidence biases, corresponding to the global tendency of participants to over or underestimate confidence irrespective of metacognitive performance. Indeed, contrary to several studies which did not control for first-order performance^33–35^, we found no overconfidence in errors nor underconfidence in correct responses except in the recollection task. One possibility is that the confidence biases previously reported in schizophrenia also stem from first-order deficit differences. Furthermore, contrary to previous behavioral results showing a positive link between false alarms and positive symptoms or proneness to hallucinations^36–38^, our sample of patients had comparable rates of false alarms compared to healthy controls in the visual detection task. They committed more false alarms in the memory tasks, interpreted as false recognitions, but no relationships were found between rates of false alarms and PANSS positive score (see Supplementary Information).

At a conceptual level, the framing of our memory tasks in terms of familiarity and recollection processes may be questionable. Indeed, although our recognition memory tasks shared some features with usual familiarity and recollection tasks (in particular, the testing questions, which are respectively context-independent and context-dependent), there was no delay between encoding and testing phases, as we manipulated task difficulty with a variable lag. Therefore, one may consider our tasks to reflect working memory, which is also known to involve familiarity and recollection processes^39^. To our knowledge, no study assessed metacognition related to short-term memory in schizophrenia. At first sight, our results seem to be in contradiction with the study by Berna and colleagues^8^, which reported impaired metamemory in schizophrenia in a long-term (autobiographical) memory task while controlling statistically for first-order performance. Yet, if our results are construed as evidence for preserved “short-term” metamemory in schizophrenia, the contradiction might be only apparent. A full taxonomy of metamemory processes is beyond the scope of the present study, and developing new paradigms to assess metacognitive performance in distinct subdomains of memory while controlling for first-order performance is one of the numerous challenges the metacognitive field is facing^40^.

With these technical and conceptual considerations in mind, we can contextualize our findings and assess their clinical relevance with caution, considering the relatively small sample size on which they are based. The level of depression was slightly higher than what is usually reported in previous samples of stabilized outpatients (range: [3.2–3.9]^41^). The average BCIS composite score was in the upper range of those reported in previous studies, including individuals with stabilized schizophrenia [4.5–8.6]^42^. The average SSTICS total score was slightly above those reported in previous studies (range: [19–26])^43^, and the average PANSS total score suggested weak symptom intensity, between “borderline mentally ill” and “mildly ill”^44^. This suggests that the present results may not necessarily be generalizable to individuals with lower cognitive insight.

Our protocols focus on “in-the-moment” metacognition^45^, i.e., confidence in trial-by-trial decisions, also known as “local” metacognition, as opposed to more “global” evaluations^46–48^. Metacognitive evaluations have been construed as hierarchically organized, where aggregated local judgments give rise to global self-beliefs about one’s performance within a cognitive task or domain^49^. Interestingly, it has been shown that global metacognitive evaluations can be altered independently from the local monitoring processes^50^. Yet, as recently discussed^51^, both local and global measures of metacognition may give an incomplete picture of metacognitive abilities from a clinical perspective. This concern is corroborated by the analyses exploring the association between metacognitive sensitivity and symptoms of schizophrenia. We found a lack of association between cognitive insight measured by SSTICS and metacognitive sensitivity, thus suggesting that the general tendency to report cognitive problems in everyday life was independent of the ability to calibrate confidence just after a decision on a trial-by-trial basis. The picture was less clear regarding the association between metacognitive sensitivity and another dimension of cognitive insight, measured with the BCIS, which evaluates the ability to examine and question beliefs and interpret experiences. We found a marginally positive association with inconclusive evidence between these two constructs, therefore requiring additional explorations. A previous study reported a lack of association between BCIS and local metacognition with moderate evidence^30^. We also found a lack of association between positive symptoms and local metacognition. Therefore, our study did not confirm the significant association between synthetic metacognition (drawing upon a broad range of social, executive, linguistic, and metacognitive processes, such as the Metacognitive Assessment Scale^52^) and positive symptoms previously reported^53^. Our results suggest that the tendency to experience hallucinations or delusion is not related to the ability to calibrate confidence. We found a negative association with inconclusive evidence between metacognitive sensitivity and negative symptoms and disorganization, therefore requiring additional explorations. Perceptual reasoning assessed with WAIS matrix subtest scores were positively correlated with metacognitive sensitivity, as reported previously^30^. The need for paradigms that do justice to the breadth of the metacognition construct, i.e., including more cognitive domains like theory of mind which has been associated with medication adherence^54^ and larger timescales, is now becoming acknowledged by the field.

### Supplementary information

SI

## Data Availability

The present design, hypotheses, and analyses were preregistered prior to data collection and analysis (https://osf.io/k4p79). Data and analysis scripts are available online (https://gitlab.com/nfaivre/metaface_scz_public).
